# Examining mediators of associations of food insecurity and being bullied with suicide among in-school adolescents in Eswatini: a cross-sectional study

**DOI:** 10.1038/s41598-023-28767-x

**Published:** 2023-01-30

**Authors:** Mfundi President Sebenele Motsa, Hung-Yi Chiou, Mattia Sanna, Maswati S. Simelane, Fortunate S. Shabalala, Yi-Hua Chen

**Affiliations:** 1 Ph.D. Program in Global Health and Health Security, College of Public Health, Taipei Medical University, Taipei, Taiwan; 2 School of Public Health, College of Public Health, Taipei Medical University, 250 Wu-Hsing St., Taipei, 110 Taiwan; 3 PhD Program in Global Health, College of Public Health, Taipei Medical University, Taipei, Taiwan; 4 Department of Statistics and Demography, Faculty of Social Sciences, University of Eswatini, Kwaluseni, Eswatini; 5 Department of Community Health Science, Faculty of Health Sciences, University of Eswatini, Mbabane, Eswatini; 6 Research Centre for Health Equity, College of Public Health, Taipei Medical University, Taipei, Taiwan; 7 Institute of Population Health Sciences, National Health Research Institutes, Zhunan Town, Miaoli County 35053 Taiwan; 8 Behavioral Research and Innovations Unit, Educational Youth Empowerment, Manzini, Eswatini

**Keywords:** Neuroscience, Psychology, Medical research

## Abstract

We examined the potential mediating roles of anxiety and loneliness on the association of concurrent food insecurity (FI) and being bullied (BB) with suicidal behavior (SB) in Eswatini, a lower-middle-income country. We used data from the Global School-based Student Health Survey (GSHS; *N* = 3264), which employed a two-stage cluster sampling: first, 25 schools were selected based on the proportionate probability of enrollment; second, classes were randomly selected. A self-reported 84-item GSHS questionnaire was used to collect data for students aged 13–17 years. FI was measured by requesting students to recall how often they went hungry because of a lack of food at home in the 30 days before the study. Multiple logistic regressions and binary mediation function was applied to examine mediating factors of SB. The prevalence of SB, FI, and BB among adolescents was 27.5%, 7.7%, and 30.2%, respectively. Moreover, the relationship between FI and BB with SB was partly (approximately 24%) mediated by anxiety and loneliness. Our results highlight the mediating roles of anxiety and loneliness in suicidal adolescents who experience FI and BB. In conclusion, interventions for alleviating SB in high-risk adolescents experiencing FI and BB should also be aimed at ameliorating anxiety and loneliness.

## Introduction

Suicidal behavior (SB), particularly among adolescents, is a serious public health concern and leads to consequences such as emotional distress and economic loss (i.e., loss of productive capacity). Suicide is the leading cause of death globally in youth aged 15–19 years^1^. Although the prevalence of suicide is comparable in both developed and developing countries (12.7 vs. 11.2 per 100,000 individuals), the proportion of suicide deaths (75.5%) is higher in developing countries than in developed (24.5%) countries^2^.

Sociodemographic factors, such as younger age, female sex^3^, school grade^4^, substance use^5^, lifestyle factors^6^, food insecurity (FI), and being bullied (BB), are well-known risk factors for SB among adolescents^7^. Studies evaluating the association of concurrent FI and being bullied (BB) with SB are lacking. The burden of FI and BB is high in sub-Saharan Africa (SSA)^8,9^. The evaluation of concurrent FI and BB in Eswatini represents a crucial addition to the literature because adolescents who encounter adversities exhibit mental health problems^10^. Therefore, the identification of possible pathways can lead to informed policy decisions. In addition, the coexistence of FI and BB in Eswatini remains underexplored. In Eswatini, the level of FI increased from 18.2% in 2015 to 29% in 2022^7,9^. Adolescents who endure FI may experience bullying and other issues, leading to mental health problems, such as SB^7^. Previous studies have examined the coexistence of FI and other behaviors; however, concurrent FI and BB remains unexplored^10^*.* Therefore, understanding the cumulative association of FI and BB on SB is essential for advancing suicidology research.

According to a global mental health survey, FI is associated with poor mental health and psychosocial stressors across regions^11^. FI is a family issue, and BB occurs mainly in schools; both influence an individual’s mental health and possibly even worsen SB outcomes. Moreover, among adolescents, BB is associated with SB^12^ in that it further worsens an individual’s capacity to deal with their suicidal symptoms. The amount of research on BB among in-school adolescents has been larger in developed countries^13,14^ than in SSA countries^15^. However, a Nigerian study on secondary school students demonstrated a significant positive relationship between BB and suicidal ideation^16^.

Because FI and BB are positively associated with SB^17^ and the risk of anxiety and loneliness^18^, studies have suggested that anxiety and loneliness may be the risk factors for SB^19^. In addition, empirical evidence suggests that internalizing behaviors, such as anxiety and loneliness, play a crucial role in the association of BB and SB^20,21^. Consequently, research on how these factors affect one another and how they lead to SB is warranted. A study evaluating the applicability of the interpersonal theory of suicide (IPTS) to adolescents contended that it may provide a basis for organizing suicide research^22^. The IPTS suggests that burdensomeness, thwarted belongingness, and capability of suicide are the key predictors of SB^23,24^. In this framework, FI and BB are viewed as burdensomeness characteristics that interact with anxiety and loneliness (viewed as thwarted belongingness)^25^, which in turn lead to SB. Some empirical studies have applied these theories to explain the main predictors of SB^25–27^. We hypothesized that the association of concurrent FI and BB with SB is mediated by anxiety and loneliness during adolescence, which represents a transitional and vulnerable period of an individual’s life.

Although some studies have focused on the relationship between indicators of adolescent social adversities (FI or BB) and SB^17,28^, studies evaluating the association of concurrent FI and BB with SB in SSA adolescents are limited. Several cross-sectional studies have reported that accumulated childhood adversities are positively associated with increased SB risk^10,29^; however, this evidence is not applicable to FI and BB, especially among adolescents. A study reported that the overlap of FI and BB is associated with psychological distress and not SB in particular^30^. In SSA, the prevalence of adolescent adversities (FI and BB) is high, and there is a lack of evidence on the potential mediating role of anxiety and loneliness in SSA. Therefore, seeking to understand the pathways linking these factors with SB in low-and-middle-income (LMIC) settings is warranted to elucidate this research question.

In Eswatini, most studies have focused on infectious diseases. However, the roles of anxiety and loneliness in the association of FI and BB with SB as well as the underlying mechanisms in this region warrant investigation because such an investigation can establish a basis for future suicide prevention strategies in SSA. Therefore, in this study, we examined the possible mediating association of anxiety and loneliness on the relationship of FI and BB with SB among adolescents in Eswatini and investigated the combined association of FI and BB on SB, anxiety, and loneliness in this population.

## Methods

### Data source

In this cross-sectional study, we used data from the 2013 Global School-based Health Survey (GSHS) conducted in Eswatini. The details of the GSHS design, methodology, and sampling methods are described elsewhere^31^. In brief, the GSHS is a collaborative effort led by the World Health Organization (WHO), United Nations International Children’s Emergency Fund (UNICEF), United Nations International Education Scientific and Cultural Organization (UNESCO), and the Joint United Nations Programme on HIV/AIDS (UNAIDS), with technical assistance from the U.S. Centers for Disease and Control (CDC), to estimate the burden of health behaviors and protective factors among in-school adolescents.

Data for the primary survey were collected through self-administered questionnaires containing 84 sociodemographic and behavioral items; questionnaires were administered during one regular class period. The students reported their responses to each question on a computer-scannable answer sheet^32^.

### Sampling and sample size

The survey employed a two-stage cluster sampling design, where schools were selected based on the proportionate probability of enrollment in the first stage. Classes were then randomly selected in the second stage. In total, 25 schools were selected, and all (100%) schools completed the survey. All students in the selected classes were eligible to join the study^32^.

In total, 3793 students were sampled; of them, 3680 completed the questionnaire (overall response rate = 97%). We included 3264 students aged 13–17 years who responded to the questions related to the main variables, namely FI, BB, loneliness, anxiety, and SB. We also assessed the differences in sociodemographic characteristics; only those who responded to all the questions regarding the main variables mentioned above (*N* = 3264) were included, whereas those who had any missing information related to these variables (n = 416) were excluded. No significant differences were observed in the demographic variables (e.g., age and sex) between the participants who were included and excluded.

### Outcome variables

The main outcome variable SB was defined as a combination of suicidal ideation, suicide planning, and suicide attempt^33^. Suicidal ideation was measured based on the following question: “During the past 12 months, did you ever seriously consider attempting suicide?” Moreover, suicide planning was analyzed using this question: “During the past 12 months, did you make a plan about how you would attempt suicide?” The possible responses for both these questions were “yes” and “no.” Suicide attempt was measured by asking the following question: “During the past 12 months, how many times did you actually attempt suicide?” The possible responses were “0,” “1,” “2,” “3,” “4,” “5,” and “6 or more times.” For analytical purposes, we used binary recoding for the suicidal ideation and suicidal planning variables (yes and no), and for suicidal attempts, we recoded one or more suicide attempts as “yes” and zero attempts as “no”^34,35^. Furthermore, if any of the three items was “yes,” then the SB variable was “yes” (1); otherwise, it was “no” (0). The Cronbach alpha for this assessment was 0.74.

### Main independent variables

FI and BB were the main independent variables in this study. As described in the literature^36,37^, FI was measured by reviewing the responses to this question: “During the past 30 days, how often did you go hungry because there was not enough food at home?” The possible responses ranged from 1 (*never*) to 5 (*always*); therefore, through binary coding, we coded 1 (*never*) to 3 (*sometimes*) as “no” and 4 (*most of the time*) to 5 (*always*) as “yes”.

BB was measured based on the following question: “During the past 30 days, how were you bullied most often?” The possible responses were as follows: (1) “I was not bullied during the past 30 days”; (2) “I was hit, kicked, pushed, shoved around, or locked indoors”; (3) “I was made fun of because of my race, nationality, or color”; (4) “I was made fun of because of my religion”; (5) “I was made fun of with sexual jokes, comments, or gestures”; (6) “I was left out of activities on purpose or completely ignored”; (7) “I was made fun of because of how my body or face looks”; and (8) “I was bullied in some other way.” All responses for which option (1) was selected were coded as “No,” whereas those with the options (2)–(8) selected were coded as “yes”.

We further divided the coded responses for FI and BB variables into four categories: “yes” for FI and BB = both FI and BB existed, “yes” for FI alone = only FI existed, “yes” for BB alone = only BB existed, and “no” for FI and BB = neither FI nor BB existed.

### Mediator variables

Anxiety and loneliness were considered as potential mediators in the study. Loneliness and anxiety were respectively measured according to the following questions: “During the past 12 months, how often have you felt lonely?” and “During the past 12 months, how often have you been so worried about something that you could not sleep at night?” The responses ranged from 1 (*never*) to 5 (*always*), and consistent with the GSHS analysis, we coded the responses from 1 (*never*) to 3 (*sometimes*) as “no” and from 4 (*most of the time*) to 5 (*always*) as “yes”^38^.

### Covariates

We selected lifestyle, interpersonal, and demographic factors as the covariates on the basis of another study that evaluated their association with our main variables^39^. These factors included physical attacks (“no” or “yes”), fights (“no” or “yes”), close friendships (“no” or “yes”), truancy (“no” or “yes”), marijuana use (“no” or “yes”), parental support (“no” or “yes”), parental monitoring (“no” or “yes”), academic support (“no” or “yes”), peer support (“no” or “yes”), age (“ ≤ 14 years” or “ ≥ 15 years”) and sex (“male” or “female”).

### Statistical analysis

We employed Stata (version 15; Stata Corp., College Station, TX, USA) for all statistical analyses. The “svy” command in Stata was used to account for complex survey design and sampling probabilities across clusters and strata. Participant distributions according to SB, anxiety, and loneliness were assessed using the chi-square test. We then fitted logistic regression models to estimate odds ratios (ORs) and their 95% confidence intervals (CIs) to assess the association of FI and BB with anxiety, loneliness, and SB. The significance level was set at 0.05, and all tests used were two-tailed. Finally, we applied a two-step mediation analysis for binary outcomes to identify the total effect of FI and BB on SB as well as the association of FI and BB acting through the mediators anxiety and loneliness. We followed Baron and Kenny’s recommendations for mediation analysis^40^ and reported bootstrapping and the results of Sobel’s test with bias-corrected estimates^41^.

### Ethical considerations

The GSHS complied with the standards of human subjects protection, where the WHO local office was responsible for application for the protocol review of the initial survey and approved by the Eswatini National Health Research Review Board (formerly Swaziland Scientific and Ethics Committee). Informed consent was obtained from each participant’s parents before questionnaire administration. The Ministry of Health and Ministry of Education and Training of Eswatini (formerly Swaziland) gave permission to conduct the study within the schools before the survey. All the methods were performed in accordance with the declarations and guidelines of Helsinki. The GSHS datasets were de-identified and made publicly accessible for analysis from the CDC and WHO websites^31,42^.

## Results

### Distribution of SB, anxiety, and loneliness in association with FI and BB

Tables 1 and 2 present the distribution of concurrent FI and BB, SB, anxiety, and loneliness among the participants. Of 3,264 adolescents, 897 (27.5%) exhibited SB, 897 (27.5%) were lonely, and 278 (8.5%) experienced anxiety. Only 3.5% of the participants reported concurrent FI and BB, with 4.3% and 27.5% reporting only FI and BB, respectively.

**Table 1:** Distribution of suicidal behavior in association with food insecurity and being bullied.

Characteristics	Total (*N* = 3264)	Suicidal behavior		p
	n (%)	Yes (n = 897)	No (n = 2367)	
Food insecurity or being bullied				
Food insecurity and being bullied	111 (3.5)	61 (6.8)	50 (2.2)	< 0.000***
Being bullied only	876 (27.4)	309 (35.0)	567 (24.4)	
Food insecurity only	139 (4.3)	42 (4.7)	97 (4.2)	
None	2138 (64.8)	485 (53.6)	1653 (69.2)	
Physical attack				
Yes	984 (31.6)	347 (40.3)	654 (28.2)	< 0.000***
No	2184 (68.4)	535 (59.7)	1685 (71.8)	
Fight				
Yes	564 (17.8)	217 (24.8)	347 (15.1)	< 0.000***
No	2692 (82.2)	677 (75.2)	2015 (84.9)	
No friends				
Yes	535 (16.4)	184 (20.6)	351 (14.8)	0.001**
No	2713 (83.6)	703 (79.4)	2010 (85.2)	
Truancy				
Yes	458 (14.5)	192 (21.9)	266 (11.5)	< 0.000***
No	2740 (85.5)	682 (78.1)	2058 (88.5)	
Marijuana				
Yes	230 (6.9)	100 (11.2)	130 (5.2)	< 0.000***
No	2947 (93.1)	764 (88.8)	2183 (94.8)	
Protective factors				
Academic support				
Yes	1077 (34.5)	288 (33.4)	789 (34.9)	0.32
No	2148 (65.5)	597 (66.6)	1551 (65.1)	
Peer support				
Yes	933 (28.6)	229 (26.0)	704 (29.6)	0.09
No	2296 (71.4)	661 (74.0)	1635 (70.4)	
Parental support				
Yes	1311 (41.6)	312 (36.5)	999 (43.6)	0.004*
No	1848 (58.4)	548 (63.5)	1300 (56.4)	
Parental monitoring				
Yes	1025 (32.3)	268 (30.6)	757 (32.9)	0.37
No	2175 (67.7)	609 (69.4)	1566 (67.1)	
Demographics				
Sex				
Male	1524 (48.2)	405 (47.8)	1119 (48.4)	0.81
Female	1712 (51.8)	478 (52.2)	12,234 (51.6)	
Age				
≤ 14 years	702 (23.2)	187 (22.0)	515 (23.6)	0.43
≥ 15 years	2521 (76.8)	695 (78)	1826 (76.4)	
Educational level				
Secondary school	2121 (71.7)	661 (79.8)	1460 (68.6)	0.004**
High school	1101 (28.3)	221 (20.2)	880 (31.4)	

*p < 0.05, **p < 0.01, ***p < 0.005.

**Table 2:** Distribution of anxiety and loneliness in association with food insecurity and being bullied.

Characteristics	Anxiety		p	Loneliness		p
	Yes (n = 278)	No (n = 2986)		Yes (n = 897)	No (n = 2367)	
Food insecurity or being bullied						
Food insecurity and being bullied	34 (13)	77 (2.6)	< 0.000**	61 (6.8)	50 (2.2)	< 0.000**
Being bullied only	124 (44.9)	752 (25.8)		309 (35)	567 (24.2)	
Food insecurity only	18 (6.6)	121 (4.1)		42 (4.7)	97 (4.2)	
None	102 (35.6)	2036 (67.5)		485 (53.6)	1653 (69.2)	
Physical attack						
Yes	327 (39.9)	753 (28.7)	0.002**	122 (37.7)	879 (31.0)	0.003**
No	512 (60.1)	1672 (71.3)		208 (62.3)	2012 (69.0)	
Fight						
Yes	72 (27.3)	492 (17)	< 0.000**	81 (24.8)	483 (17.0)	0.003**
No	205 (72.7)	2487 (83)		256 (75.2)	2436 (83.0)	
No friends						
Yes	61 (22.2)	474 (15.9)	< 0.003**	97 (28.8)	438 (15.0)	< 0.000**
No	215 (77.8)	2498 (84.1)		238 (71.2)	2475 (85.0)	
Truancy						
Yes	64 (23.8)	394 (13.6)	< 0.002**	67 (20.7)	391 (13.8)	< 0.001**
No	207 (76.2)	2533 (86.4)		263 (79.3)	2477 (86.2)	
Marijuana						
Yes	33 (12.0)	197 (6.4)	< 0.000***	44 (13.3)	186 (6.2)	< 0.001**
No	233 (88)	2714 (93.6)		281 (86.7)	2666 (93.8)	
Protective factors						
Academic support						
Yes	73 (27.7)	1004 (35.1)	0.01*	85 (26.5)	992 (35.2)	< 0.000**
No	201 (72.3)	1947 (64.9)		248 (73.5)	1900 (64.6)	
Peer support						
Yes	78 (27.7)	855 (28.7)	0.73	90 (27.0)	843 (28.8)	0.49
No	198 (72.3)	2098 (71.3)		243 (73.0)	2053 (71.2)	
Parental support						
Yes	82 (30.5)	1229 (42.6)	0.001***	119 (36.8)	1192 (42.1)	< 0.02*
No	187 (69.5)	1661 (57.4)		204 (63.2)	1644 (57.9)	
Parental monitoring						
Yes	67 (26.1)	958 (32.8)	0.10	86 (26.5)	939 (32.9)	< 0.01*
No	202 (73.9)	1973 (67.2)		243 (73.5)	1932 (67.1)	
Demographics						
Sex						
Male	111 (43.1)	1413 (48.7)	0.20	156 (48.9)	1368 (48.1)	0.85
Female	163 (56.9)	1549 (51.3)		176 (51.1)	1536 (51.9)	
Age						
≤ 14 years	39 (15.4)	663 (23.9)	0.20	61 (19.7)	641 (23.6)	0.19
≥ 15 years	235 (84.6)	2286 (76.1)		270 (80.3)	2251 (76.4)	
Educational level						
Secondary school	157 (63.9)	1964 (72.5)	0.07	195 (65.4)	1926 (72.5)	0.007**
High school	116 (36.1)	985 (27.5)		136 (34.6)	965 (27.5)	

*p < 0.05, **p < 0.01, ***p < 0.005.

Adolescents with SB tended to have experienced both FI and BB, have been physically attacked, have been in a fight, have been truant, have no friends, have used marijuana, and have no parental support (all p < 0.05). Moreover, adolescents with anxiety tended to have no academic or parental support (both p < 0.05). Finally, adolescents with loneliness tended to have no parental monitoring (p < 0.05).

### Factors associated with SB

Table 3 presents the results of the univariate and multivariable logistic regression for variables associated with SB. After adjustment for covariates, concurrent FI and BB [adjusted OR (aOR) = 3.16, 95% CI 1.74–5.74] and BB alone (aOR = 1.48, 95% CI 1.24–1.79) were significantly associated with an increased risk of SB compared with not having FI or BB. Furthermore, having been physically attacked (aOR = 1.32, 95% CI 1.07–1.61), having been in a fight (aOR = 1.46, 95% CI 1.19–1.79), having no friends (aOR = 1.36, 95% CI 1.11–1.65), having been truant (aOR = 1.81, 95% CI 1.46–2.26), and having used marijuana (aOR = 1.78, 95% CI 1.25–2.54) were significantly associated with increased SB risks compared with their counterparts.

**Table 3:** Association of food insecurity and being bullied on suicidal behavior.

Characteristics	Suicidal behavior	
	cOR (95% CI)	aOR (95% CI)
Food insecurity and being bullied		
Food insecurity and being bullied	4.03 (2.27–7.16)***	3.16 (1.74–5.74)***
Being bullied only	1.85 (1.59–2.15)***	1.48 (1.24–1.79)***
Food insecurity only	1.42 (0.98–2.07)	1.19 (0.79 –1.80)
None	1	1
Physical attack		
Yes	1.71 (1.40–2.09)***	1.32 (1.07–1.61)*
No	1	1
Fight		
Yes	1.84 (1.53–2.23)***	1.46 (1.19–1.79)**
No	1	1
No friends		
Yes	1.49 (1.21–1.81)**	1.36 (1.11–1.65)**
No	1	1
Truancy		
Yes	2.15 (1.86–2.51)***	1.81 (1.46–2.26)***
No	1	1
Marijuana		
Yes	2.29 (1.74–3.09)***	1.78 (1.25–2.54)**
No	1	1
Peer support		
No	1.20 (0.97–1.49)	1.10 (0.84–1.43)
Yes	1	1
Academic support		
No	1.06 (0.93–1.22)	0.91 (0.79–1.03)
Yes	1	1
Parental support		
No	1.34 (1.20–1.62)***	1.21 (1.03–1.44)
Yes	1	1
Parental monitoring		
No	1.11 (0.86–1.43)	0.92 (0.69–1.23)
Yes	1	1
Sex		
Female	1.02 (0.84–1.24)	1.23 (0.95–1.60)
Male	1	1
Age		
≥ 15 years	0.91 (0.72–0.99)**	1.08 (0.85–1.38)
≤ 14 years	1	1

*cOR* crude odds ratio, *aOR* adjusted odds ratio, *CI* confidence interval.

*p < 0.05, **p < 0.01, ***p < 0.005.

Adjusted for all other listed factors.

### Factors associated with anxiety and loneliness

Table 4 presents the results of the univariate and multivariable logistic regression for anxiety and loneliness risk factors. After adjustment for covariates, concurrent FI and BB (aOR = 7.26, 95% CI 4.33–12.2), BB alone (aOR = 2.80, 95% CI 2.23–3.52), and FI alone (aOR = 3.02, 95% CI 1.73–5.27) were significantly associated with an increased risk of anxiety compared with not having FI or BB. Moreover, having been in a fight (aOR = 1.30, 95% CI 1.04–1.62), having no friends (aOR = 1.42, 95% CI 1.12–1.79), having no parental support (aOR = 1.34, 95% CI 1.03–1.74), and being female (aOR = 1.63, 95% CI 1.12–2.38) were significantly associated with an increased risk of anxiety compared with their counterparts.

**Table 4:** Association of food insecurity and being bullied on anxiety and loneliness.

Characteristics	Anxiety		Loneliness	
	cOR (95% CI)	aOR (95% CI)	cOR (95% CI)	aOR (95% CI)
Food insecurity and being bullied				
Food insecurity and being bullied	9.49 (6.05–14.8)***	7.26 (4.33–12.2)***	6.51 (4.28–9.88)***	5.71 (3.70–8.83)***
Being bullied only	3.29 (2.59–4.19)***	2.80 (2.23–3.52)***	2.29 (1.65–3.20)***	2.22 (1.59–3.07)***
Food insecurity only	3.01 (1.88–4.83)***	3.02 (1.73–5.27)***	3.38 (1.76–6.50)***	3.39 (1.56–7.38)***
None	1	1	1	1
Physical attack				
Yes	1.62 (1.32–2.11)**	1.15 (0.84–1.58)	1.34 (1.13–1.60)***	1.02 (0.80–1.31)
No	1	1	1	1
Fight				
Yes	1.84 (1.44–2.15)***	1.30 (1.04–1.62)***	1.60 (1.21–2.12)***	1.28 (0.87–1.92)
No	1	1	1	1
No friends				
Yes	1.50 (1.17–1.93)***	1.42 (1.12–1.79)**	2.29 (1.72–3.05)***	2.31 (1.73–3.07)***
No	1	1	1	1
Truancy				
Yes	1.98 (1.33–2.96)**	1.28 (0.81–2.02)	1.63 (1.28–2.08)***	1.01 (0.72–1.42)
No	1	1	1	1
Marijuana				
Yes	1.98 (1.30–3.04)**	1.29 (0.78–2.13)	2.34 (1.67–3.27)***	1.48 (0.93–2.34)
No	1	1	1	1
Peer support				
No	1.04 (0.77–1.49)	0.90 (0.58–1.40)	1.09 (0.83–1.43)	0.86 (0.66–1.12)
Yes	1	1	1	1
Academic support				
No	1.41 (1.08–1.84)*	1.11 (0.89–1.39)	1.52 (1.26–1.84)***	1.34 (1.06–1.70)*
Yes	1	1	1	1
Parental support				
No	1.69 (1.32–2.17)***	1.34 (1.03–1.74)*	1.25 (1.04–1.50)*	1.11 (0.91–1.36)
Yes	1	1	1	1
Parental monitoring				
No	1.39 (0.92–2.09)	1.28 (0.84–1.96)	1.36 (1.09–1.70)*	1.10 (0.90–1.35)
Yes	1	1	1	1
Sex				
Female	1.25 (0.87–1.79)	1.63 (1.12–2.38)*	0.97 (0.70–1.35)	1.12 (0.77–1.62)
Male	1	1	1	1
Age				
≥ 15 years	0.57 (0.32–1.03)	1.66 (0.91–3.02)	0.79 (0.55–0.90)**	1.23 (0.82–1.82)
≤ 14 years	1	1	1	1

*cOR* crude odds ratio, *aOR* adjusted odds ratio, *CI* confidence interval.

*p < 0.05, **p < 0.01, ***p < 0.005.

Adjusted for all other listed factors.

Regarding loneliness, after the covariates were adjusted for, having both FI and BB (aOR = 5.71, 95% CI 3.70–8.83), BB alone (aOR = 2.22, 95% CI 1.59–3.07), and FI alone (aOR = 3.39, 95% CI 1.56–7.38) were significantly associated with an increased risk of loneliness compared with not having FI or BB. Finally, having no friends (aOR = 2.31, 95% CI 1.73–3.07) and having no academic support (aOR = 1.34, 95% CI 1.06–1.70) were associated with increased risk for loneliness compared to their counterparts.

### Mediating role of anxiety and loneliness

**Figure 1:**
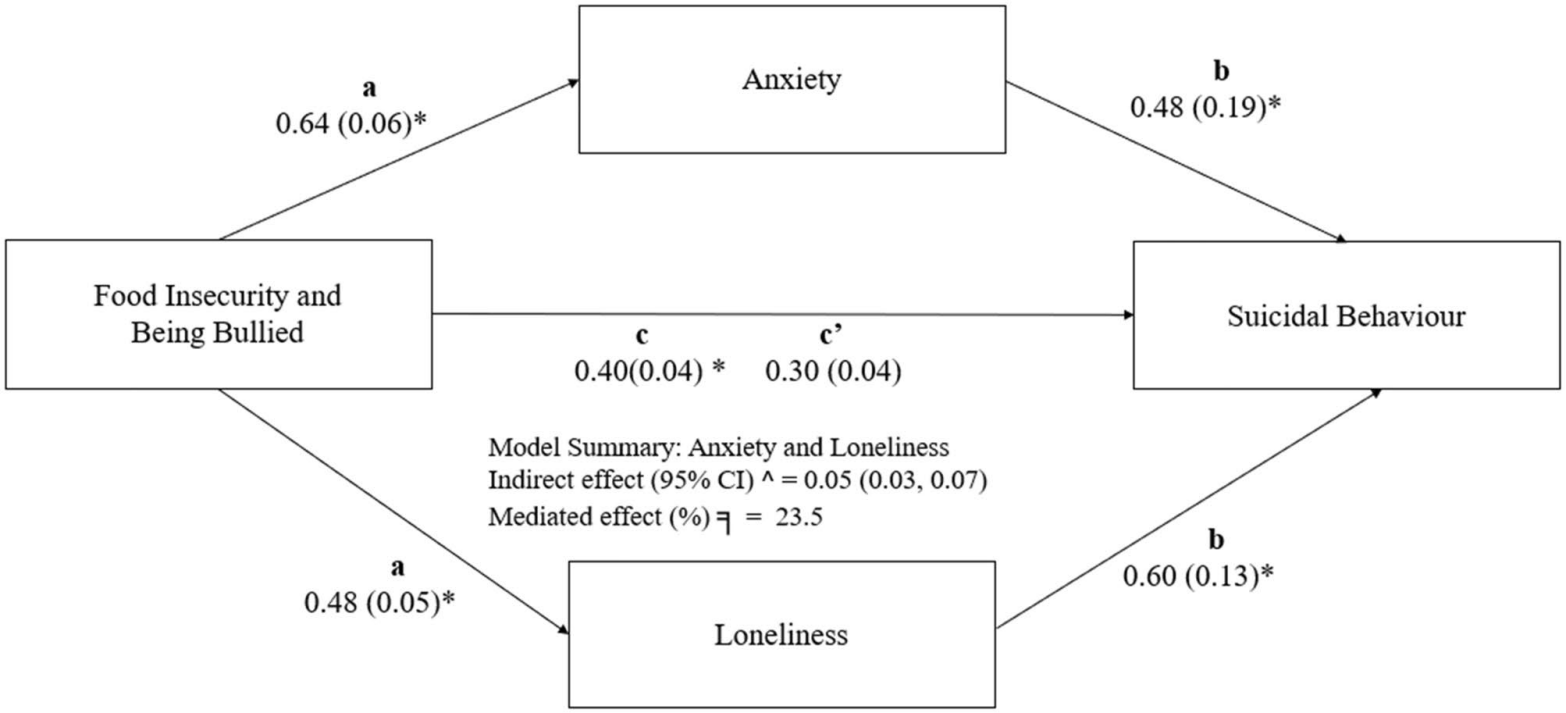
Mediating role of anxiety and loneliness symptoms on the relationship of food insecurity and being bullied on suicidal behavior. Values outside the parentheses are unstandardized path coefficients, whereas those in the parentheses are standard errors. Path (**a**) represents the coefficient for the effects of the main variables on the mediators (anxiety and loneliness), Path (**b**) the coefficient for the effects of the mediators (anxiety and loneliness) on suicidal behaviour after the main independent variables are adjusted for, and Path (**c′**) the effects of the main independent variable (food insecurity and being bullied) on suicidal behavior when the mediators (anxiety and loneliness) are adjusted for. *CI* confidence interval. ^ = bias-corrected confidence interval. ╕ = proportion of total effect mediated. *p < 0.05.

Figure 1 displays the results of the potential mediating roles of anxiety and loneliness in the association of concurrent FI and BB with SB. In Step 1, FI and BB were found to be significantly associated with SB (Path c, B = 0.40). Next, concurrent FI and BB were noted to be significantly associated with anxiety (mediator 1) and loneliness (mediator 2; Path a; B = 0.64 and 0.48, respectively). In Step 3, we found that when FI and BB were controlled for, anxiety and loneliness were significantly associated with SB (Path b, B = 0.48 and 0.60, respectively).

The indirect effect of concurrent food insecurity and being bullied through anxiety and loneliness was significant (0.05, 95% CI [0.03, 0.07]), suggesting that anxiety and loneliness mediates the association between concurrent food insecurity and being bullied and suicidal behavior (the proportion of the total mediated was 23.5%). The results were supported by the goodness of fit index, with a standardized root mean square of residuals of 0.085.

## Discussion

To the best of our knowledge, this is the first study to confirm the mediating roles of anxiety and loneliness in the association of concurrent FI and BB with SB among adolescents in Eswatini, an SSA region. It is also the first population-based study to investigate the association of concurrent FI and BB with SB in adolescents, and the results indicated that concurrent FI and BB is significantly associated with an increased risk of SB. Our finding is consistent with that of a study^43^ reporting that childhood difficulties increase the risk of SB. Moreover, FI is a biological and psychological stressor and, therefore, leads to SB^44,45^. Our results indicated that adolescents who experience concurrent FI and BB is associated with risk for anxiety and loneliness, a result similar to that of another study^45^. However, experiencing FI only was not associated risk for SB, which is in contrast to the results of an SSA-based study^46^. We also discovered that adolescents who experience anxiety and loneliness exhibit an increased risk of SB, which is consistent with the results of previous studies^44,47^. Feeling anxious and lonely can exacerbate other mental health issues associated with SB, especially in adolescents, who do not share their problems with anyone and consequently become more anxious.

The significant association of concurrent FI and BB with SB decreased significantly when anxiety and loneliness were introduced into the model. This finding suggests that our variables substantially affected each other, indicating that adolescents who experienced FI and BB may have developed anxiety and loneliness, which in turn resulted in SB. Therefore, interventions aimed at reducing anxiety and loneliness may eventually reduce SB risk. Moreover, the proportion of total mediated risk was 23.5%, which suggests the importance of focusing on the mediation pathways noted in the current study for SB prevention and intervention among adolescents. Other factors that may influence SB risk in adolescents include psychological conditions and stressful life events, such as interpersonal difficulties (peer rejection) and family conflicts (parental divorce). Our findings accord with those of another study, which found that loneliness is a mediator for social exclusion and adolescent mental health issues^48^; nonetheless, the influence of loneliness on SB is unique. Our results may be explained based on the observation that individuals may ideate suicide if they feel they are a burden and lack a sense of belonging^23^.

We discovered that BB alone was associated with risk for anxiety, loneliness, and SB; however, FI alone was associated with risk for anxiety and loneliness but not for SB. It could be that there was under reporting and also possibilities of few cases because these issues are rare as there are community strengthening activities of sustainable development goals in achieving zero hunger by 2030^9^. Further studies are required to clarify and update the association between FI and SB, especially in SSA. Our findings support the hypothesis that FI^49^ and BB^14,50^ are associated with risk for mental health problems in adolescents. Although adolescence is a critical stage of child mental health development^51^, the co-occurrence of childhood adversities including FI^7,18^ and BB^52^ can increase the likelihood of SB, anxiety, and loneliness. Our findings demonstrated that the combined difficulties endured by adolescents both at the school level (BB) and family level (FI) during their development affect their mental health^43,53^. In adolescents, increased exposure to difficult situations can eventually lead to stress, which can in turn result in SB^54^.

Notably, in our sample, FI and BB prevalence was 3.5% and 30.2%, respectively, and these values are lower than those reported in Ghana (57.8% and 41.3%, respectively)^55,56^. Moreover, in the current study, the prevalence of anxiety and loneliness was 8.5% and 27.5%, respectively, which are lower than those in an SSA-based study (12.2% for anxiety)^57^ and in Morocco (19.8% for loneliness)^57^. SB prevalence was 27.5%, which is similar to that in other LMIC countries, where the prevalence of suicide ideation, planning, and attempt was 20.4%, 23.7%, and 19.3%, respectively^58^. Although the prevalence of FI, BB, and anxiety was lower in Eswatini than in the SSA region on average, loneliness and SB prevalences were high, which is a cause for concern and warrants further research on the contextual factors related to this burden. The observed variation in prevalence could be linked to geographic and cultural differences.

### Strengths and limitations

Our study has several strengths. First, this study is the first to analyze a nationally representative survey from SSA with a high response rate (97%) to assess the mediating role of anxiety and loneliness in the association of FI and BB with SB. Second, we employed a robust selection method that enables the possible generalization of the results to adolescents enrolled in schools. Finally, our results confirmed the synergistic effect of concurrent FI and BB on loneliness, anxiety, and SB in high-risk groups.

This study also has some limitations. The current findings should be interpreted with caution because suicide is a sensitive issue and the SB items in the GSHS might have been interpreted differently by the students even though the instruments were validated and used across countries. Because our data were exclusively based on self-reports, our results may have been affected by social desirability bias. Moreover, because of the cross-sectional study design, causality and examining the sequence of events of the factors could not be inferred; therefore, future studies with an improved design are warranted. For example, FI is not only a family issue but also affects countries; therefore, school nutrition programs and government transfers may reduce the levels of FI among adolescents. However, a questionnaire-based study measured the influence of some factors, such as diet, on FI among adolescents^59^. In addition, our study used single item in measuring key variables including FI. Although similar design was also utilized in other GSHS studies^60–62^, future studies could consider robust scales like the household food insecurity access scale (HFIAS) for evaluation*.* We cannot rule out the possibility of reverse causality because of the cross-sectional study design and inconsistency of the measurement tools (FI and BB occurrences within the 30 days before questionnaire administration were evaluated, whereas the occurrences of other key variables were measured in the 12 months before the study). Because the survey tools were used previously in in-school adolescent behavior research, future studies should use more precise survey instruments (e.g., the Patient Health Questionnaire-9). In addition, employing a longitudinal design could help to infer causal relations. We were unable to consider family-level and school-level characteristics appropriately because of the nature of our dataset.

### Recommendations

The current findings may aid in devising and establishing suicide intervention and prevention efforts in schools for adolescents. Our findings indicate that concurrent FI and BB affects mental health and, therefore, should be considered when developing physical and mental health programs for school children. Anxiety and loneliness partially mediated the association of concurrent FI and BB with SB, suggesting that interventions for reducing anxiety and loneliness levels may affect SB also. To reduce the role of concurrent FI and BB and the risk of SB, food availability issues should be addressed and antibullying programs must be implemented.

This study highlighted that a lack of close friends is associated with the risks of anxiety, loneliness, and SB among adolescents; this result emphasizes the role of interpersonal relationships in this age group. Finally, our results indicate the immediate need to tailor contextual, comprehensive SB prevention interventions by incorporating the significant factors observed in this study for Eswatini adolescents.

## Conclusion

We discovered that anxiety and loneliness mediate the association of concurrent FI and BB with SB. The combined role of FI and BB on anxiety, loneliness, and SB was also revealed. Although this study has some limitations, we expanded the current knowledge by (1) examining the combined role of FI and BB in SB development and (2) testing for mediators of this relationship, both by using a large sample from SSA. In so doing, we demonstrated a transfer of the methodological approach for low-resource settings in child psychiatry and psychology. Future studies should confirm our results and use precise data collection instruments (e.g., Beck Scale for Suicide Ideation and Generalized Anxiety Disorder-7), incorporating depression and life events in analyses, as well as using a longitudinal design.

## Data Availability

The data for this manuscript is readily available and can be accessed on the World Health Organization Non-communicable disease data repository; https://extranet.who.int/ncdsmicrodata/index.php/catalog/central.
